# Epidemiological Perspectives: A Four-Year Insight Into Hepatitis C Surveillance in the Kingdom of Saudi Arabia

**DOI:** 10.7759/cureus.52646

**Published:** 2024-01-20

**Authors:** Ali Almajid, Hassan Albarbari, Ali Bazroon, Hashim Al-Awami, Rahaf Aljurayyad, Razan Albadran, Zainab Alkhamis, Haider Alomair, Yamama Aljishi

**Affiliations:** 1 Department of Internal Medicine, King Fahad Specialist Hospital, Dammam, SAU; 2 College of Medicine, Imam Mohammad Ibn Saud Islamic University, Riyadh, SAU; 3 College of Medicine, Imam Abdulrahman Bin Faisal University, Dammam, SAU; 4 College of Medicine, Gulf Medical University, Ajman, ARE

**Keywords:** moh, saudi arabia, surveillance, hcv, hepatitis c virus

## Abstract

Introduction

Hepatitis C is a viral disease caused by the hepatitis C virus (HCV), a member of the *Flaviviridae* family. This compact, enveloped RNA virus possesses a positive single-stranded genome and can be transmitted through various means, including blood exposure, sexual contact, and vertical transmission. The disease is associated with significant morbidity and mortality, imposing substantial costs on the healthcare system. In Saudi Arabia, HCV is a notifiable disease; however, there is a scarcity of recent reports on HCV trends in the country. This study aims to provide updated insights into the infection patterns of HCV across demographics, regions, and genders in Saudi Arabia.

Methods

A retrospective analysis was conducted to investigate the epidemiological trends of HCV infection in Saudi Arabia. Data were obtained from the Saudi Ministry of Health (MOH), encompassing the timeframe from 2019 to 2022. A descriptive analysis of HCV infection, organized by year, age group, and gender, was conducted using the data reported to the MOH.

Results

Between 2019 and 2022, there was a significant decrease of 56.9% in the overall rate of hepatitis C cases in Saudi Arabia. The rate dropped from 9.94 to 4.29 cases per 100,000 people during this period. Males consistently had higher reported cases compared to females, although there was a notable decline in cases for both genders from 2019 to 2022. The highest incidence of HCV was found in individuals aged 45 years and above. However, there was a decline in cases among this age group, with the number dropping from 2,195 cases in 2019 to 946 cases in 2022. In terms of regional variations, Riyadh, Makkah, Jeddah, Alsharqiya, and Taif had the highest incidence of HCV cases. Some regions experienced an increase in cases between 2021 and 2022, particularly Jeddah, Taif, and Al-Ahsaa.

Conclusion

This study reveals a significant reduction in reported HCV cases in Saudi Arabia from 2019 to 2022. However, gender disparities persist, with males having a higher number of reported cases. There is also a notable decline in HCV cases among children and adolescents, which can be attributed to preventive measures. The findings emphasize the importance of region-specific strategies, as certain areas, such as Riyadh, Makkah, Jeddah, Alsharqiya, and Taif, continue to have a high number of reported cases. Proactive measures, surveillance, and public awareness campaigns remain crucial in addressing HCV as a significant public health challenge in the Kingdom.

## Introduction

Hepatitis C is a viral disease caused by the hepatitis C virus (HCV), which belongs to the *Flaviviridae* family. It is a compact, enveloped RNA virus with a positive single-stranded genome [1]. HCV is highly transmissible and can be transmitted through various means, including exposure to infected blood through intravenous drug use, vertical transmission from mother to child, blood transfusion, sexual contact, or organ transplantation [2]. Sexual transmission of HCV is increasingly recognized among homosexual individuals infected with human immunodeficiency virus (HIV) [3]. If left untreated, HCV infection can lead to chronic infection and negative health outcomes. However, with the development of direct-acting antiviral medications, HCV can now be effectively cured [4].

HCV infection is a significant global health issue, with approximately 500,000 individuals dying from HCV-related diseases each year [5]. The global prevalence of active HCV infection is estimated to be around 1% [6]. In response to this public health concern, the Centers for Disease Control and Prevention (CDC) recommends one-time screening for all adults aged 18-75 years and individuals of any age who have been exposed to risk factors [7].

In Saudi Arabia, HCV infection has been a notifiable disease since 1990 [8]. Previous studies have indicated an average incidence rate of 124 per 100,000 individuals in the country [9]. Given the substantial impact of HCV infection on affected individuals and the healthcare system, it is important to understand the trends of HCV infection in Saudi Arabia. However, there is a relative scarcity of recent reports detailing these trends. Therefore, the objective of the current study is to provide an updated analysis of the prevailing patterns of HCV infection among the Saudi population. This analysis will include an examination of infection trends across different age groups, regions, and genders, aiming to contribute valuable insights to the understanding of HCV epidemiology in the country.

## Materials and methods

An observational, cross-sectional study was conducted to investigate the epidemiological trends of HCV infection in Saudi Arabia. Data were obtained from the Saudi Ministry of Health (MOH), covering the period from 2019 to 2022 [8]. The MOH collected data on nationwide cases of HCV, and the confirmation of these cases relied on HCV antibody testing. Importantly, the reported instances of HCV do not undergo differentiation based on infection status, whether characterized as acute or chronic. A descriptive analysis of HCV infection, organized by year, age group, and gender, using simple statistics, was conducted with the data reported to the MOH. Approval from the Institutional Review Board (IRB) was deemed unnecessary for the present study design, given that the data were sourced from the Saudi MOH online registry.

## Results

Between 2019 and 2022, there was an overall decrease in the rate of hepatitis C cases in Saudi Arabia (Table 1).

**Table 1:** Total cases of HCV and its incidence per 100,000 population. HCV, hepatitis C virus

Year	2019	2020	2021	2022
Total cases	3,402	2,031	1,960	1,464
Anti-HCV newly diagnosed patients per 100,000 population, N (%)	9.94 (0.00994%)	5.8 (0.0058%)	5.75 (0.00575%)	4.29 (0.00429%)

**Figure 1:**
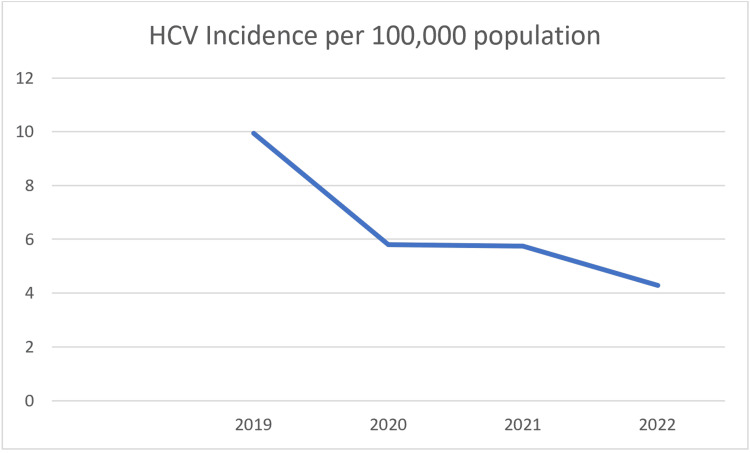
Rate of reported HCV cases per 100,000 population. HCV, hepatitis C virus

In 2019, there were 3,402 reported cases, corresponding to a rate of 9.94 per 100,000 people. By 2022, this rate had decreased by 56.9% to 4.29 per 100,000 people (Figure 1).

**Figure 2:**
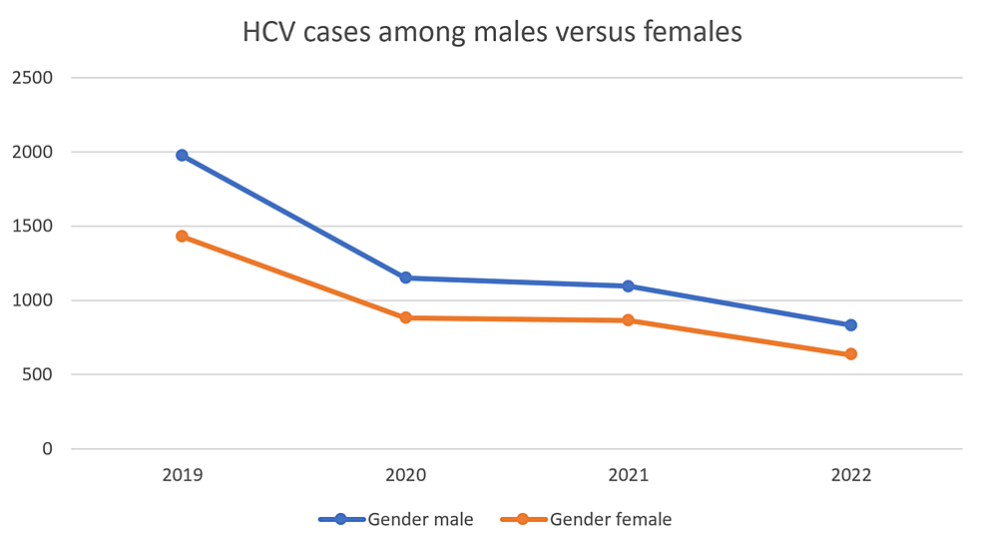
Rate of reported HCV cases among males versus females between 2019 and 2022. HCV, hepatitis C virus

The rates of reported cases of hepatitis C were consistently higher among males than females throughout the study period. In 2019, there were 1,973 cases among males (57%) and 1,429 cases among females (42%). In 2022, the total number of cases decreased to 1,464, with 832 cases among males (56.8%) and 632 cases among females (43%) (Figure 2).

**Figure 3:**
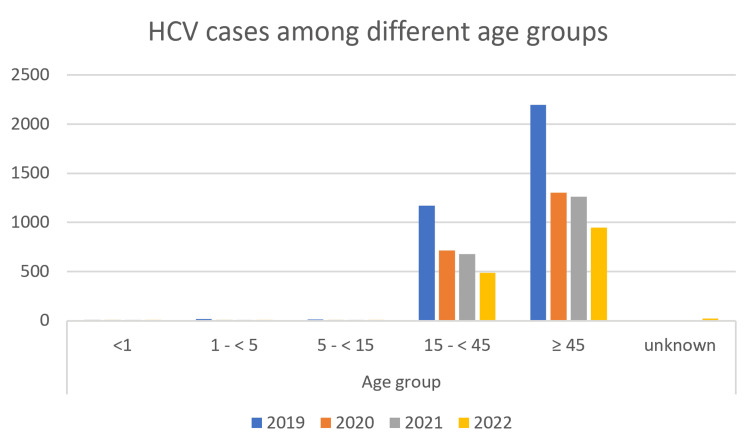
Rate of reported HCV cases between 2019 and 2022 based on age. HCV, hepatitis C virus

The peak incidence of reported HCV cases was observed in individuals aged 45 years and above. In 2019, there were 2,195 cases in this age group, which decreased to 946 cases in 2022. On the other hand, the occurrences of HCV among adolescents and children were significantly lower. In the pediatric age group below 15 years, only 36 cases were reported in 2019, further decreasing to nine cases in 2022 (Figure 3).

**Figure 4:**
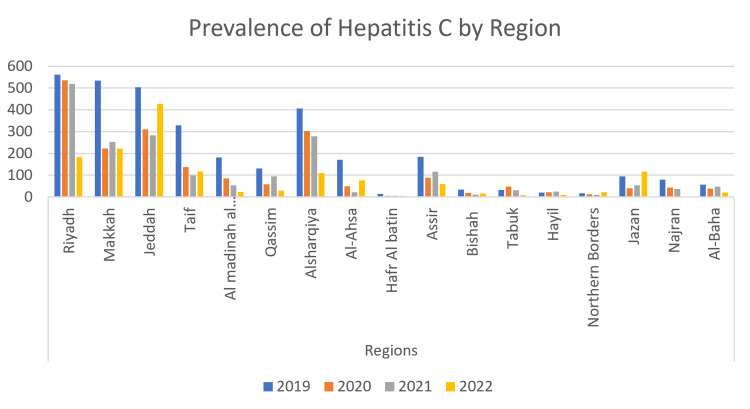
Rate of reported HCV cases among different regions in Saudi Arabia between 2019 and 2022. HCV, hepatitis C virus

The incidence of reported HCV cases varied among different regions in Saudi Arabia (Figure 4). The highest incidence was noted in Riyadh, Makkah, Jeddah, Alsharqiya, and Taif, in descending order. Notably, Jeddah, Taif, and Al-Ahsaa experienced an increase in HCV cases between 2021 and 2022. In Jeddah, the rate increased from 14.4% in 2021 to 29.2% in 2022. Similarly, in Al-Ahsaa, the rate rose from 1% in 2021 to 5% in 2022, and in Taif, it increased from 5% to 8%.

## Discussion

The present study was conducted to elucidate the trends in the incidence and prevalence of hepatitis C in Saudi Arabia in recent years, providing the most up-to-date data currently available on reported cases and corresponding rates of HCV in Saudi Arabia.

The result highlighted four pivotal findings. Firstly, cases of HCV infection are on a decline among the Saudi population. Secondly, the rate of HCV infection is consistently higher among males than females. Thirdly, the rate of HCV infection was on the rise on certain areas of the Kingdom of Saudi Arabia such as Jeddah, Taif, and Al-Ahsaa. Fourthly, the rate of HCV infection is significantly higher among the population aged 45 years and older.

A study conducted over 11 years reported that the highest prevalence of HCV cases was observed in Al Baha and Jeddah, reaching 0.32%, while the lowest prevalence was noted in Jazan at 0.016% [9]. Conversely, we observed the highest incidence of HCV cases was noted in Riyadh, Jeddah, and Alsharqiya. This could be attributed to the more developed healthcare structure and wider testing in these more developed provinces. The estimated prevalence rates for individuals aged less than 15 years and adults were calculated at 0.012% and 0.202%, respectively [9]. In line with our findings, we found a significantly higher rate of HCV among adults compared to the pediatric age group.

Three investigations centered on serological markers in blood donors from Eastern and Central Saudi Arabia. These studies revealed a decrease in prevalence rates over time and highlighted a greater prevalence of these markers among donors who are not Saudi in contrast to Saudi donors. Additionally, the research demonstrated a correlation between advancing age and an elevated prevalence of anti-HCV, aligning with our observations [10-12]. In a previous report, it has been noted that HCV is most prevalent in adults born between 1949 and 1965, accounting for approximately three-fourths of all cases in the United States of America. It has been recommended to screen individuals born during that time frame, as delaying identification and, consequently, care will negatively impact health outcomes [13]. Additionally, we observed a higher incidence of HCV among males. It has been indicated that men who have sex with other men are at a higher risk of being infected by HCV [14]. Therefore, these populations should receive special attention when implementing a screening program.

In the results of blood screening from donors in Jeddah, a 1.7% prevalence of HCV was revealed. Another study conducted in Riyadh reported a 1.1% prevalence of HCV [15-16]. In a study conducted by Memish et al. focused on viral hepatitis surveillance, an annual mean incidence was documented at 78.4 per 100,000 individuals within the population served by the National Guard Health Affairs (NGHA) hospitals in the Eastern, Western, and Central regions of the country. Notably, the study revealed a substantial 30% reduction in HCV incidence over an eight-year period [17]. The reported prevalence of HCV in the Arab world is similar to that observed in the Kingdom of Saudi Arabia. Notably, Egypt exhibits an exceptionally high prevalence (18.1%, and up to 40% in specific regions), likely attributed to historical mass treatment campaigns [18-22].

Globally, surveillance data from Canada spanning the period of 1998 to 2012 elucidated a pervasive decrease in hepatitis C rates among females. Notably, this overall decline was contrasted with an exception among individuals aged 25 to 29 years, where an increase was observed. The most conspicuous reduction was discernible in the demographic of females aged 10 to 14 years, albeit this observation was tempered by the constrained number of cases within this age group. Upon excluding this particular age cohort, the most substantial reduction manifested among females aged 30 to 39 years [23]. In line with our research findings highlighting the elevated incidence of HCV among males, an additional study conducted in Canada in 2007 systematically analyzed the prevalence of infection, stratified across both gender and age cohorts. The results revealed a significant 64% higher prevalence of HCV among males compared to females, with rates of 0.95% and 0.61%, respectively [24].

The decline in HCV cases in Saudi Arabia can be attributed to various factors, including government initiatives, mandatory screening of blood donors, pre-marital testing, and the availability of direct-acting antiviral treatments. These interventions have contributed to the reduction in HCV transmission and improved health outcomes for individuals infected with the virus [25-28].

The projected healthcare expenditures associated with HCV in Saudi Arabia emphasize the need for continued efforts to eliminate the virus [29]. Implementing mandatory HCV screening, efficient linkage to care, and proactive initiation of DAA treatment are crucial steps toward achieving comprehensive HCV elimination and reducing the economic burden associated with the disease.

Strengths and limitations

Our study has multiple strengths points. Firstly, the study obtained its data from the MOH of Saudi Arabia, providing a comprehensive and authoritative source for nationwide cases of HCV over a recent four-year period (2019 to 2022). Secondly, the study captures a temporal perspective, allowing for the observation of changes in HCV incidence over the specified period. The present study does have limitations to be acknowledged. Firstly, the reported instances of HCV do not undergo differentiation based on infection status (acute or chronic). This lack of differentiation limits the ability to analyze the severity and persistence of HCV cases. Secondly, a detailed description of the reported cases and the treatment received was not readily available for further analysis.

## Conclusions

In conclusion, this study contributes valuable data to our understanding of HCV epidemiology in Saudi Arabia. It highlights the progress made in reducing HCV incidence but also emphasizes the need for ongoing efforts to combat the disease. Proactive measures, including mandatory HCV screening, timely access to HCV care, and the use of DAA treatments, are crucial to further mitigate the burden of HCV in the country. Continued surveillance and research efforts are essential to monitor trends, identify emerging challenges, and inform public health strategies for the comprehensive elimination of HCV in Saudi Arabia.

## References

[1] Morozov VA, Lagaye S (2018). Hepatitis C virus: morphogenesis, infection and therapy. World J Hepatol.

[2] Schillie S, Wester C, Osborne M, Wesolowski L, Ryerson AB (2020). CDC recommendations for hepatitis C screening among adults-United States. MMWR Recomm Rep.

[3] Myers T, Allman D, Xu K (2009). The prevalence and correlates of hepatitis C virus (HCV) infection and HCV-HIV co-infection in a community sample of gay and bisexual men. Int J Infect Dis.

[4] Muir AJ, Naggie S (2015). Hepatitis C virus treatment: is it possible to cure all hepatitis C virus patients?. Clin Gastroenterol Hepatol.

[5] (2024). WHO. Hepatitis C Fact Sheet No 164. https://www.who.int/news-room/fact-sheets/detail/hepatitis-c.

[6] Maheshwari A, Ray S, Thuluvath PJ (2008). Acute hepatitis C. Lancet.

[7] Owens DK, Davidson KW, Krist AH (2020). Screening for hepatitis C virus infection in adolescents and adults: US Preventive Services Task Force Recommendation Statement. JAMA.

[8] (2024). Hepatitis C cases in KSA. https://od.data.gov.sa/Data/en/dataset/hepatitis-c-cases-in-ksa.

[9] Madani TA (2007). Hepatitis C virus infections reported in Saudi Arabia over 11 years of surveillance. Ann Saudi Med.

[10] Abdelaal M, Rowbottom D, Zawawi T, Scott T, Gilpin C (1994). Epidemiology of hepatitis C virus: a study of male blood donors in Saudi Arabia. Transfusion.

[11] Shobokshi OA, Serebour FE, Al-Drees AZ, Mitwalli AH, Qahtani A, Skakni LI (2003). Hepatitis C virus seroprevalence rate among Saudis. Saudi Med J.

[12] El-Hazmi MM (2004). Prevalence of HBV, HCV, HIV-1, 2 and HTLV-I/II infections among blood donors in a teaching hospital in the Central region of Saudi Arabia. Saudi Med J.

[13] Smith BD, Morgan RL, Beckett GA (2012). Recommendations for the identification of chronic hepatitis C virus infection among persons born during 1945-1965. MMWR Recomm Rep.

[14] (2018). EASL recommendations on treatment of hepatitis C 2018. J Hepatol.

[15] Shobokshi OA, Serebour FE, Skakni LI (2003). Hepatitis C genotypes/subtypes among chronic hepatitis patients in Saudi Arabia. Saudi Med J.

[16] Alshahrani MY, Alamri AM, Alshahrani AS (2021). Prevalence of selected blood-borne infectious diseases among voluntary blood donors in abha, Saudi Arabia. Southeast Asian J Trop Med Public Health.

[17] Memish ZA, Knawy BA, El-Saed A (2010). Incidence trends of viral hepatitis A, B, and C seropositivity over eight years of surveillance in Saudi Arabia. Int J Infect Dis.

[18] Darwish MA, Raouf TA, Rushdy P, Constantine NT, Rao MR, Edelman R (1993). Risk factors associated with a high seroprevalence of hepatitis C virus infection in Egyptian blood donors. Am J Trop Med Hyg.

[19] Darwish MA, Faris R, Clemens JD, Rao MR, Edelman R (1996). High seroprevalence of hepatitis A, B, C, and E viruses in residents in an Egyptian village in The Nile Delta: a pilot study. Am J Trop Med Hyg.

[20] Frank C, Mohamed MK, Strickland GT (2000). The role of parenteral antischistosomal therapy in the spread of hepatitis C virus in Egypt. Lancet.

[21] Rao MR, Naficy AB, Darwish MA, Darwish NM, Schisterman E, Clemens JD, Edelman R (2002). Further evidence for association of hepatitis C infection with parenteral schistosomiasis treatment in Egypt. BMC Infect Dis.

[22] Darwish MA, Faris R, Darwish N (2001). Hepatitis c and cirrhotic liver disease in the Nile delta of Egypt: a community-based study. Am J Trop Med Hyg.

[23] Payne E, Totten S, Archibald C (2014). Hepatitis C surveillance in Canada. Can Commun Dis Rep.

[24] Remis RS (2024). Section 4: Modelling the Incidence and Prevalence of Hepatitis C Infection and its Sequelae in Canada, 2007 - Discussion. Community Acquir Infect Div Cent Commun Dis Infect Control Heal Agency Canada.

[25] Abdo AA, Sanai FM, Al-Faleh FZ (2012). Epidemiology of viral hepatitis in Saudi Arabia: are we off the hook?. Saudi J Gastroenterol.

[26] Gosadi IM (2019). National screening programs in Saudi Arabia: overview, outcomes, and effectiveness. J Infect Public Health.

[27] Alotaibi AS, Shamas N, Ansari UU, Sanai FM, Alshahrani A, Fathelrahman AI, Aseeri MA (2021). Impact of drug use policy on the appropriate use of direct acting antiviral agents for Hepatitis C in Saudi Arabia. J Pharm Bioallied Sci.

[28] Politi J, Guerras JM, Donat M, Belza MJ, Ronda E, Barrio G, Regidor E (2022). Favorable impact in hepatitis C-related mortality following free access to direct-acting antivirals in Spain. Hepatology.

[29] Altraif I (2018). Can hepatitis C virus be eliminated by 2030? Saudi Arabia as an example. Saudi Med J.

